# Feasibility of continuous bronchoscopy during exercise in the assessment of large airway movement in healthy subjects

**DOI:** 10.1152/japplphysiol.00746.2023

**Published:** 2024-04-25

**Authors:** Zander J. Williams, Christopher M. Orton, Justin L. Garner, Ley T. Chan, Anand Tana, Pallav L. Shah, Michael I. Polkey, Thomas Semple, James H. Hull

**Affiliations:** ^1^Department of Respiratory Medicine, Royal Brompton Hospital, London, United Kingdom; ^2^National Heart and Lung Institute, Imperial College, London, United Kingdom; ^3^Department of Radiology, Royal Brompton Hospital, London, United Kingdom; ^4^Division of Surgery and Interventional Science, Institute of Sport, Exercise and Health (ISEH), University College London, London, United Kingdom

**Keywords:** airways, breathing, bronchoscopy, exercise, trachea

## Abstract

Excessive dynamic airway collapse (EDAC) is a recognized cause of exertional dyspnea arising due to invagination of the trachea and/or main bronchi. EDAC is typically assessed by evaluating large airway movement with forced expiratory maneuvers. This differs from the respiratory response to exercise hyperpnea. We aimed to evaluate large airway movement during physical activity, with continuous bronchoscopy during exercise (CBE), in healthy subjects and compare findings with resting bronchoscopic maneuvers and imaging techniques. Twenty-eight individuals were recruited to complete two visits including treadmill-based CBE, to voluntary exhaustion, and cine magnetic resonance imaging (MRI) with forced expiratory maneuvers at rest. Twenty-five subjects [aged 29 (26–33) yr, 52% female] completed the study (*n* = 2 withdrew before bronchoscopy, and one was unable to tolerate insertion of bronchoscope). The majority (76%) achieved a peak heart rate of >90% predicted during CBE. The procedure was prematurely terminated in five subjects (*n* = 3; elevated blood pressure and *n* = 2; minor oxygen desaturation). The CBE assessment enabled adequate tracheal visualization in all cases. Excessive dynamic airway collapse (tracheal collapse ≥50%) was identified in 16 subjects (64%) on MRI, and in six (24%) individuals during resting bronchoscopy, but in no cases with CBE. No serious adverse events were reported, but minor adverse events were evident. The CBE procedure permits visualization of large airway movement during physical activity. In healthy subjects, there was no evidence of EDAC during strenuous exercise, despite evidence during forced maneuvers on imaging, thus challenging conventional approaches to diagnosis.

**NEW & NOTEWORTHY** This study demonstrates that large airway movement can be visualized with bronchoscopy undertaken during vigorous exercise. This approach does not require sedation and permits characterization of the behavior of the large airways and the tendency toward collapse during upright, ambulatory exercise. In healthy individuals, the response pattern of the large airways during exercise appears to differ markedly from the pattern of airway closure witnessed during forced expiratory maneuvers, assessed via imaging.

## Graphical abstract

**Figure:**
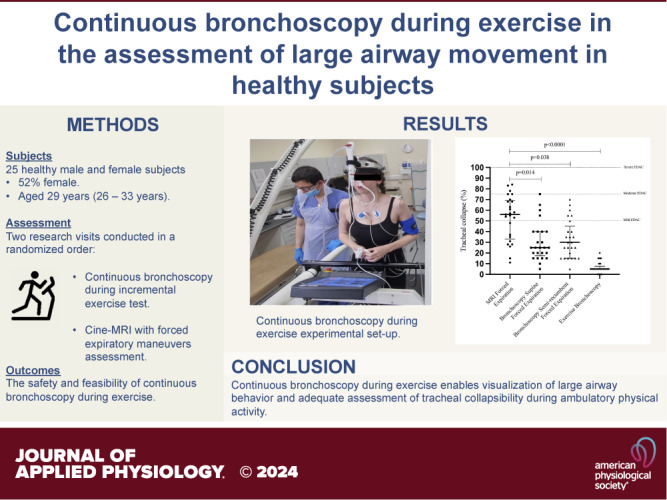

## INTRODUCTION

The large airway, defined as the section of the airway tract from the subglottis to the main bronchi, is considered to primarily function as a conduit for ventilation. The posterior segment of the large airway wall, however, partially invaginates during cough and dynamic expiratory maneuvers, reducing the cross-sectional aperture to facilitate antegrade clearance of mucus (1, 2). However, it is recognized that in some cases, this inward movement may become exaggerated and can, on occasion, completely occlude the tracheal lumen. This excessive dynamic airway collapse (EDAC) may have a deleterious impact on airflow properties and impair effective airway clearance (1).

Individuals with EDAC often also report exertional dyspnea (3). Yet, the relationship between the degree of large airway collapse, functional capability, and exercise capacity remains unclear, with only limited physiological data providing insight regarding this relationship (4). Excessive collapse of the large airways is typically characterized and diagnosed using forced or dynamic expiratory respiratory maneuvers, undertaken at rest, i.e., by the assessment of tracheal lumen changes at the end of forced expiration and most commonly, with an individual in a supine or semisupine position. Typically, a 50% or greater reduction in tracheal diameter during this type of maneuver is considered as being abnormal and in keeping with EDAC (5). It is noteworthy, however, that a reduction of up to 70%–80% in luminal diameter has previously been reported in asymptomatic, healthy individuals from an imaging-based approach to evaluation (6). The type of maneuvers typically used to diagnose EDAC, as described earlier, differ from the airway stress arising from exercise hyperpnea. Specifically, a forced expiratory maneuver generates a sudden expulsion of gas from total lung capacity to residual volume. As forced expiration commences, diaphragm relaxation and rapid contraction of expiratory musculature increase both intra-alveolar and pleural pressures. These changes in pressures drive airflow from alveolar to mouth until it reaches a point where intra-alveolar pressure becomes equal to atmospheric pressure (7). In contrast, exercise-associated hyperpnea develops with an initial, gradual increase in tidal volume via a reduction in end-expiratory lung volume and an increase in end-inspiratory volume. With increasing intensity, and in the later stages of exercise, further ventilatory demand is achieved via increases in breathing frequency (8). In health, the maximal attainable expiratory pressure measured during a forced expiratory maneuver is considerably greater than the expiratory pressures observed during exercise (145 cmH_2_O vs. 42 cmH_2_O, respectively) (9, 10).

The use of an endoscopic-based approach to evaluate airway movement during exercise has been safely performed at a laryngeal level using continuous laryngoscopy during exercise (CLE) (11). The CLE test involves the placement of a small endoscope at the level of the laryngeal inlet, which is then secured to headgear to permit continuous visualization of laryngeal movement during incremental exercise. The aim of this work was to extend this type of approach and specifically explore the safety, tolerability, and utility of continuous bronchoscopy during exercise (CBE), to visualize movement of the large airways. We aimed to characterize the large airway response to vigorous exercise in healthy subjects and to compare findings on exertion with measures taken during bronchoscopic maneuvers, i.e., those used more typically in routine clinical practice, to assess large airway movement. We also compare findings from CBE with those obtained during forced expiratory maneuvers assessed with magnetic resonance imaging (MRI). We hypothesized that the degree of large airway collapse seen following a forced expiratory maneuver would be more pronounced than any tendency to closure witnessed during physical activity.

## MATERIALS AND METHODS

### Study Design and Population

This was a single-center, pilot, prospective feasibility study, in healthy individuals, aged between 20 and 60 yr old to evaluate the safety and feasibility of CBE. Subjects were excluded if they were current smokers, pregnant, or had any significant cardiovascular, respiratory, circulatory, or neurological comorbidities. All subjects had normal baseline spirometry. Written informed consent was obtained from all subjects, and ethical approval was obtained from the London Bridge Research Ethics Committee (London, UK) (REC Reference: 19/LO/1564).

### Experimental Design

**Figure 1.**
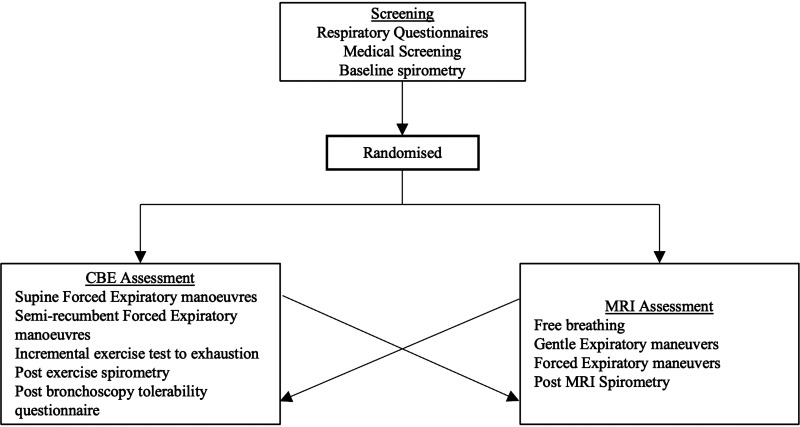
Study design and experimental procedures.

Subjects attended on two occasions. At an initial visit, subjects completed dyspnea-related questionnaires including Dyspnea-12 (12), the Medical Research Council Dyspnea Scale (13), the visual analog scale for breathlessness, and rating of perceived exertion (RPE) using a modified BORG category ratio-10 scale (range: 0–10) (14). Baseline height (cm), body mass (kg), resting blood pressure, heart rate (HR), and peripheral arterial oxygen saturation ($\text{S}{\text{p}}_{{\text{O}}_{2}}$) were recorded. Spirometry was measured (Microlab Spirometer Carefusion, Vernon Hills, IL) according to the American Thoracic Society/European Respiratory Society pulmonary function guidelines (15), and the Global Lung Initiative reference range was used. Subjects were familiarized with the study procedures and proceeded, in a random order, to either undertake bronchoscopic or MRI assessment, as detailed in the *Bronchoscopic Assessment, Including Continuous Bronchoscopy during Exercise and Dynamic Magnetic Resonance Imaging Protocol* sections below. During the subsequent visit, subjects repeated baseline spirometry and performed the alternative diagnostic assessment (Fig. 1).

### Bronchoscopic Assessment, Including Continuous Bronchoscopy during Exercise

**Figure 2.**
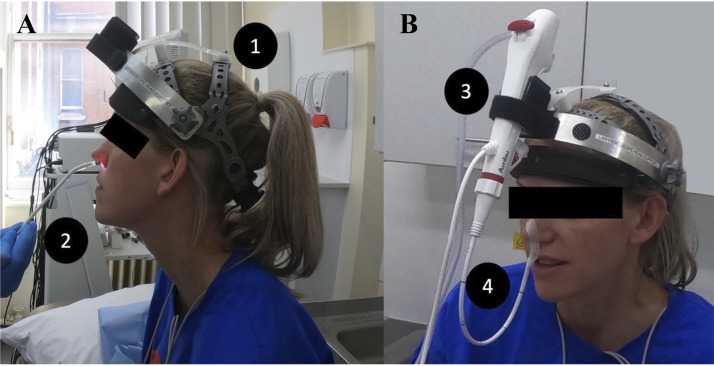
*A* and *B*: experimental setup for flexible bronchoscope. *1*) Specialist headgear, *2*) the bronchoscope is passed through the subject’s nasal cavity and vocal cords and positioned to the mid trachea. *3*) The bronchoscope control arm is secured to the specialist headgear, and *4*) the camera position is adjusted and secured with micropore surgical tape at the external nares.

Bronchoscopy was performed by a pulmonologist (C.M.O., J.L.G.). Before insertion of the bronchoscope, Instillagel (Lignocaine 2%) was applied to the nares, followed by six actuations of 2% xylocaine applied to the larynx. No sedation was administered. A single-use flexible bronchoscope (Ambu aScope 5, Ballerup, Denmark), an external diameter of 5.0 mm was passed through the nasal canal, and three aliquots of 2% lidocaine were applied under direct vision to the glottic inlet. The bronchoscope traversed the vocal cords, with four aliquots of 2% lidocaine then applied under direct vision to the trachea and main bronchi. Bronchoscopic inspection of the large airways was completed to identify areas of excessive bulging of the posterior tracheal membrane. The bronchoscope was positioned in the mid-third of the trachea to enable visualization of the distal third as determined by the experienced operator. The control body of the bronchoscope was then fastened to the subject’s head using a lightweight specialist headgear (weight = 400 g), and the bronchoscope flexible arm was secured to the nose with micropore surgical tape (Fig. 2).

**Figure A1.**
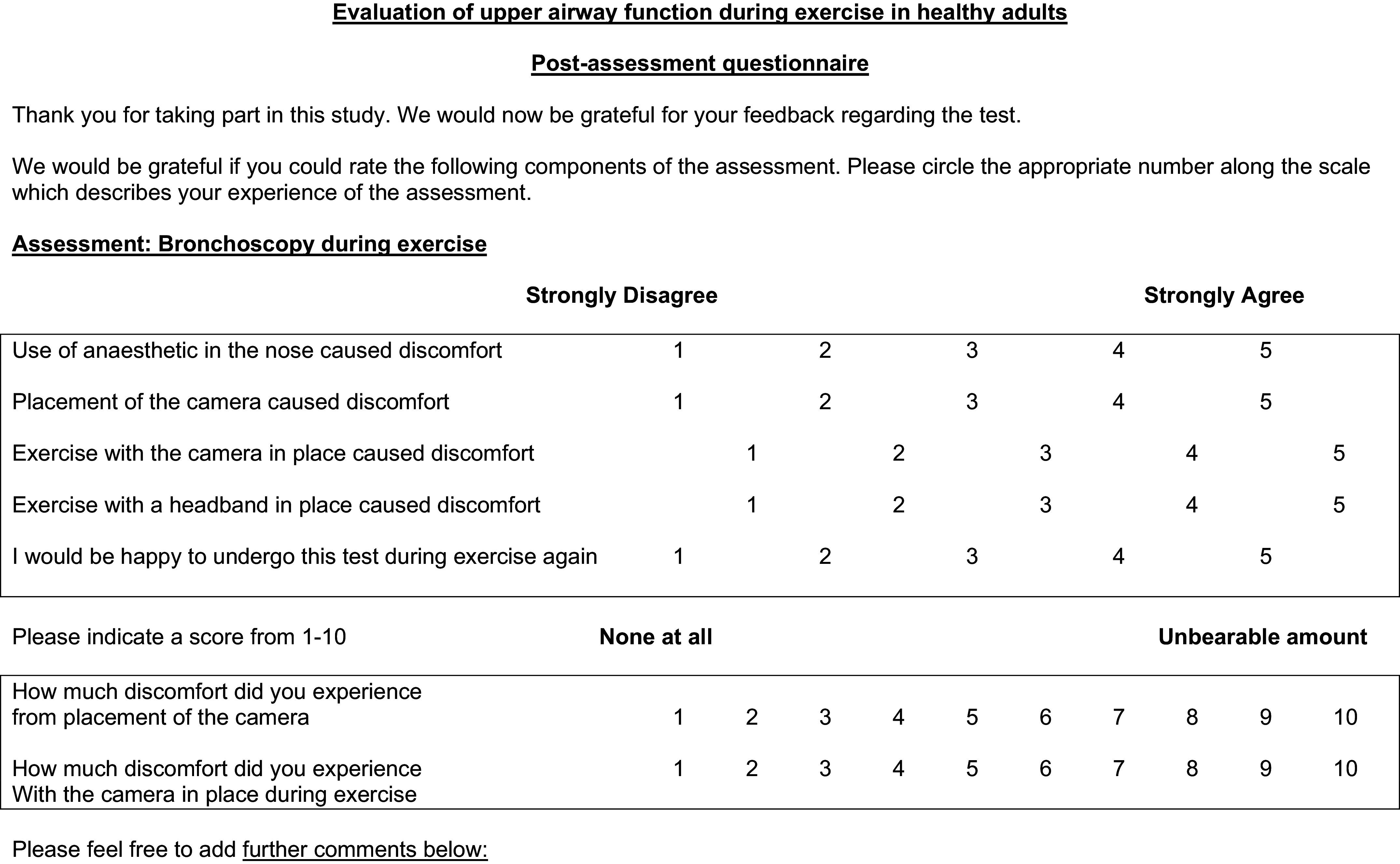
Post-tolerability feedback questionnaire for continuous bronchoscopy during exercise (CBE) assessment.

Resting assessment with forced expiratory maneuvers was then performed in the semirecumbent and supine positions with a full view of the lower trachea. Following three tidal breaths, subjects were instructed to “take a big breath in” and “blow all the way out until empty” performing a maximal forced expiratory maneuver to residual volume. Following resting assessment, subjects then moved, with bronchoscope in situ, to the treadmill for CBE testing, as described in the *Exercise Protocol for CBE Test* section below. At exercise cessation, the bronchoscope was removed and postexercise RPE assessment was completed. Finally, subjects completed a postexercise tolerability questionnaire that provided feedback on the CBE assessment as displayed in Fig. A1.

### Exercise Protocol for CBE Test

**Figure 3.**
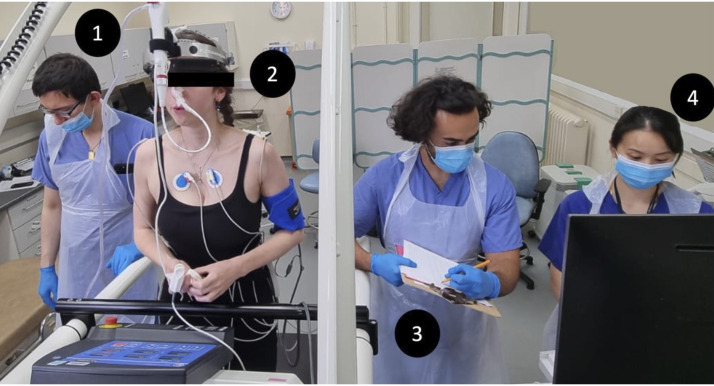
Continuous bronchoscopy during exercise laboratory setup: *1*) experienced pulmonologist (J.L.G.) reviewing airway images; *2*) subject exercising with bronchoscopy in situ; *3*) exercise physiologist recording vital sign measurements and rating of perceived exertion (RPE) scores; and *4*) clinician evaluating cardiac parameters.

The CBE test was performed on a computer-controlled treadmill (H/P/Cosmos Pluto med, H/P/Cosmos sports & medical gmbh, Germany) using a stepwise incremental protocol to volitional exhaustion (16). The subject was monitored continuously with 12-lead electrocardiogram and $\text{S}{\text{p}}_{{\text{O}}_{2}}$; blood pressure and RPE were assessed every 2 min (Fig. 3). Exercise was continued until voluntary exhaustion or terminated if abnormalities in the electrocardiogram (e.g., dysrhythmia), significant oxygen desaturation (change in $\text{S}{\text{p}}_{{\text{O}}_{2}}$ of ≥5%), or blood pressure reading of 225/110 mmHg were observed. Spirometry was repeated immediately, under 5 min, following exercise cessation and removal of the bronchoscope.

Continuous video capture of images was conducted throughout bronchoscopy assessments; respiratory maneuvers and the final minute of exercise were time-stamped for retrospective analysis by two pulmonologists (C.M.O. and J.G.) independently. The severity of EDAC was described as normal: <50% cross-sectional area (CSA) collapse; mild: 50%–75% CSA collapse; moderate >75%–<100%; and severe: CSA collapse 100% (5). The tracheal collapse data reported during bronchoscopic assessments was a mean of the two scorer’s values.

### Dynamic Magnetic Resonance Imaging Protocol

The cine-MRI protocol developed for the assessment of central airway dynamics used a series of forced expiratory maneuvers acquired at three different trachea positions. The scanning protocol was performed using a 1.5-T field strength scanner (Siemens Magnetom Avanto Fit, Erlangen, Germany) and consisted of localizer, half-Fourier acquisition single-shot turbo-spin echo images, and cine T2 true fast imaging with steady-state free precession. Subject images were obtained through the tracheobronchial tree in a supine position. During forced expiratory maneuvers, static images were acquired during 20-s sequences of the upper, middle, and lower trachea, performed with active coaching by an exercise physiologist (Z.J.W.). The top of the trachea was defined as the subglottic position, inferior to the vocal cords as the trachea dilates. The bottom position was defined as 1 cm above the carina, and the middle trachea was defined as the mid-point between the top and bottom trachea positions. Verbal instructions given during bronchoscopy and MRI-forced expiratory maneuvers assessments were identical. Overall, the acquisition time per patient, including localizers, adjustments, breathing instructions, and executing all scans, was ∼25 min (range: 18–45 min).

### Dynamic Magnetic Resonance Image Analysis

An experienced consultant radiologist (T.S.), blinded to the bronchoscopy study data, retrospectively analyzed MR images. The inspiratory and expiratory cross-sectional area (CSA) of the airway lumen was measured by tracing the inner wall of the airway with an electronic tracing tool. The inspiratory and expiratory measurements reported are the maximum and minimum area and circumference, irrespective of timing within the respiratory cycle. The percentage of expiratory lumen collapse was calculated as “percentage of luminal collapse = 100 × (1 − [luminal area at dynamic expiration/luminal area at end inspiration]).” The severity of EDAC observed in MR images was classified as per the bronchoscopy grading system.

### Safety and Tolerability

The primary outcome of the study was to evaluate the safety and feasibility of exercise bronchoscopy through adverse events, serious adverse event reporting, as per Health Research Authority guidelines (17), and feedback from a postprocedural tolerability questionnaire.

### Statistical Analysis

All statistical analyses were performed on GraphPad Prism (GraphPad Prism, v.10, San Diego, CA). The Shapiro–Wilk test was used for normality testing. Continuous, normally distributed data were presented as means and standard deviations (SD). Nonparametric data were reported as median and interquartile range (IQR). Group differences were analyzed using the Mann–Whitney *U* test, McNemar’s tests, or Chi-squared (Fisher’s exact) analysis as appropriate. A *P* value < 0.05 was considered statistically significant.

## RESULTS

### Subject Characteristics

A total of 28 healthy subjects were enrolled during the study period. The majority [*n* = 25, 52% female, aged 29 (26–33) yr] of subjects completed all study visits, characteristics presented in Table 1. Two individuals withdrew early from the study and only completed an MRI visit. One subject was unable to tolerate bronchoscope insertion through the nasal cavity due to a previous traumatic injury to the nose that resulted in a marked narrowing of the apertures. No differences were observed in male and female body mass index (BMI), and lung function was normal in all subjects.

**Table 1.** Subject characteristics

	Total (*n* = 25)	Male (*n* = 12)	Female (*n* = 13)
Age, yr	29 (26–33)	27 (26–29)	32 (25–36)
Height, cm	172 (164–183)	183 (175–185)	164 (164–167)
Weight, kg	72 (65–80)	81 (75–88)	65 (62–70)
BMI, kg/m^2^	24 (21–26)	25 (24–25)	24 (21–26)
FEV_1_, L	3.59 (3.37–4.40)	4.51 (3.94–5.10)	3.37 (3.22–3.59)
FEV_1_ % predicted	102 (95–112)	98 (93–111)	102 (96–117)
FVC, L	4.35 (4.06–5.71)	5.73 (4.87–6.26)	4.06 (3.76–4.27)
FVC % predicted	105 (98–112)	104 (101–107)	108 (98–119)

Data presented as medians (interquartile range, IQR). BMI, body mass index; FEV_1_, forced expiratory volume in one second; FVC, forced vital capacity.

The median duration of exercise was 12 min, with the majority (80%) terminating exercise due to voluntary exhaustion (Table 2). The CBE procedure was stopped prematurely in three subjects (12%) due to elevated systolic blood pressure and in two (8%) due to transient peripheral oximetry desaturation, with a median $\text{S}{\text{p}}_{{\text{O}}_{2}}$ drop of 8% (range: 7–9%). Male and female subjects achieved similar peak exercise HR values, and age-predicted maximal HR values, and exercised for similar durations (*P* = 0.11, *P* = 0.13, *P* = 0.07, respectively). The majority (76%) of subjects were able to exercise vigorously during CBE, with an end-exercise heart rate of >90% predicted. At rest, baseline BORG dyspnea and leg fatigue scores were zero. At peak exercise, median (IQR) BORG dyspnea and leg fatigue scores were similar [5 (4–7) vs. 5 (3–7), *P* = 0.28, respectively].

**Table 2.** Cardiovascular and perceptual response to continuous bronchoscopy during exercise

	Total (*n* = 25)	Male (*n* = 12)	Female (*n* = 13)
Exercise duration, min	12 (11–14)	13 (12–14)	12 (11–13)
Peak exercise heart rate, beats/min	179 (164–185)	183 (179–186)	171 (153–179)
Age-predicted maximal heart rate, %	94 (93–96)	95 (94–97)	94 (88–95)
End-exercise heart rate >90%	19 (76)	11 (92)	8 (62)
Peak exercise BORG			
Dyspnea	5 (4–7)	6 (4–7)	4 (4–7)
Leg fatigue	5 (3–7)	6 (4–8)	3 (1–6)

Data presented as median (interquartile range, IQR) or *n* (%) unless stated otherwise.

### Large Airway Assessment

**Figure 4.**
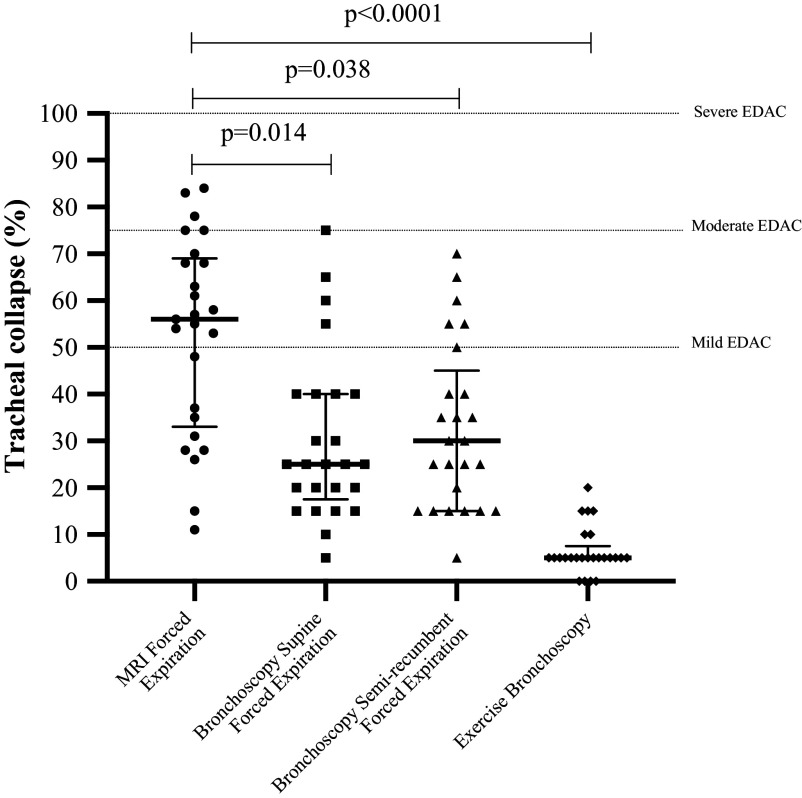
Percentage of tracheal collapse measured from maximal inspiration to end expiration during magnetic resonance imaging (MRI) and bronchoscopy. EDAC, excessive dynamic airway collapse.

The images attained during bronchoscopic visualization of the trachea and main bronchi were stable and deemed to be of adequate quality to assess change in luminal dimensions in all cases. During forced expiratory maneuvers, in the supine and semirecumbent positions, the median (IQR) percentage of tracheal membrane reduction was 25% (20%–40%) and 30% (15%–40%), respectively (Table 3). There were no differences observed between male and female subjects (both *P* > 0.5). When tracheal collapse was assessed in supine and semirecumbent positions, three (12%) and six (24%) individuals met the criteria for mild EDAC, respectively. In the supine position, one subject met the criteria for moderate EDAC. Minimal tracheal collapse was observed during peak exercise with a median (IQR) reduction in CSA of 5% (5%–5%), with no differences observed between male and female subjects (*P* > 0.05). No individuals met the criteria for EDAC (Fig. 4).

**Table 3.** Percentage of tracheal collapse measured during bronchoscopy assessments

	Total (*n* = 25)	Male (*n* = 12)	Female (*n* = 13)
Supine forced expiratory maneuvers, %	25 (20–40)	25 (19–40)	25 (20–40)
Semirecumbent forced expiratory maneuvers, %	30 (15–40)	30 (19–36)	25 (15–55)
Peak exercise, %	5 (5–5)	5 (5–5)	5 (5–10)

Data presented as median (interquartile range, IQR) unless stated otherwise.

### MRI Tracheal Circumference and Cross-Sectional Area Measurements

Tracheal circumference, cross-sectional area measurements, and percentage of luminal collapse at end inspiration and expiration are displayed in Tables 4 and 5. The percentage of CSA change at the upper, mid-trachea, and lower trachea levels were 21% (8%–27%), 45% (35%–60%), and 56% (34%–68%), respectively. There were no differences observed in percentage of luminal collapse between male and female subjects at the upper (*P* = 0.96), mid-trachea (*P* = 0.64), or lower tracheal level (*P* = 0.47). When collapse was assessed at the lower tracheal level, 44% (*n* = 11) met the criteria for mild EDAC, and 20% (*n* = 5) met the criteria for moderate EDAC.

**Table 4.** Tracheal circumference and percentage of expiratory collapse measured during MRI assessment

	Total (*n* = 25)	Men (*n* = 12)	Women (*n* = 13)
Circumference at TLC, mm			
Upper trachea	52 (46–57)	57 (56–59)	46 (41–48)
Mid-trachea	55 (50–58)	59 (56–61)	51 (42–55)
Lower trachea	51 (44–57)	57 (53–60)	44 (41–49)
Circumference during forced exhalation, mm			
Upper trachea	49 (42–55)	55 (51–57)	43 (39–46)
Mid-trachea	42 (35–51)	51 (41–55)	39 (34–42)
Lower trachea	38 (33–40)	38 (34–54)	38 (32–39)
Percentage expiratory collapse, %			
Upper trachea	5 (0–9)	6 (2–9)	5 (0–9)
Mid-trachea	18 (12–24)	15 (6–28)	19 (16–21)
Lower trachea	25 (11–35)	30 (12–37)	16 (10–30)

Data presented as median (interquartile range, IQR) or *n* (%) unless stated otherwise. Upper trachea, distal to vocal cords; mid-trachea, midpoint between upper and lower trachea; lower trachea, 1 cm above the bifurcation. TLC, total lung capacity.

**Table 5.** Tracheal cross-sectional luminal area and percentage of expiratory collapse measured during MRI assessment

	Total (*n* = 25)	Men (*n* = 12)	Women (*n* = 13)
Area at TLC, mm^2^			
Upper trachea	210 (165–250)	253 (243–259)	165 (125–180)
Mid-trachea	240 (195–260)	265 (241–280)	200 (135–240)
Lower trachea	205 (145–250)	245 (218–278)	145 (130–190)
Area during forced exhalation, mm^2^			
Upper trachea	150 (125–210)	213 (183–235)	125 (96–140)
Mid-trachea	105 (81–175)	175 (91–185)	102 (78–109)
Lower trachea	90 (56–110)	91 (76–150)	75 (56–108)
Percentage expiratory collapse, %			
Upper trachea	21 (8–26)	19 (8–25)	21 (12–26)
Mid-trachea	45 (36–60)	43 (28–61)	46 (41–50)
Lower trachea	56 (35–68)	58 (49–69)	55 (28–68)

Data presented as median (interquartile, IQR) unless stated otherwise. Upper trachea, distal to vocal cords; mid-trachea, midpoint between upper and lower trachea; lower trachea, 1 cm above the bifurcation. TLC, total lung capacity.

**Figure 5.**
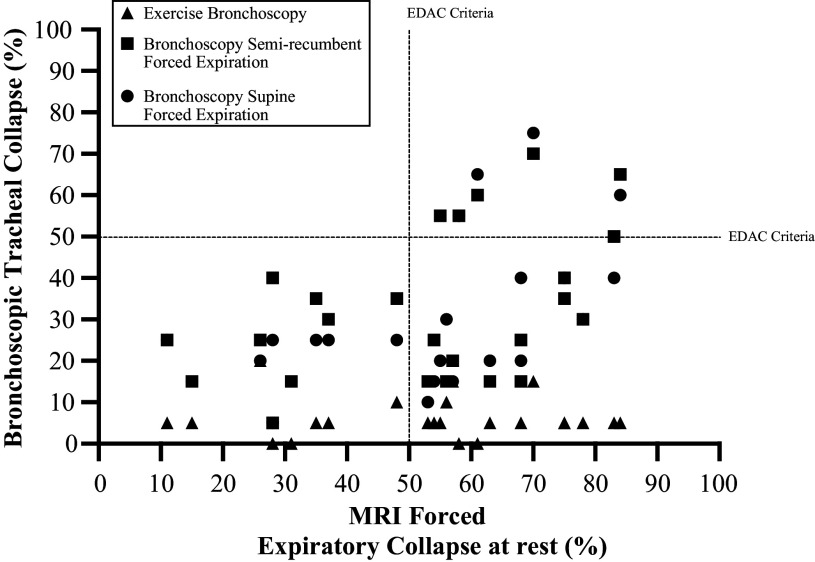
Scatter plot of the relationship between percentage collapse measured during magnetic resonance imaging (MRI) and bronchoscopy evaluation. EDAC, excessive dynamic airway collapse.

The percentage of tracheal collapse measured during MRI assessment was greater than the tracheal collapse measured during bronchoscopic forced expiratory maneuvers in the supine (*P* = 0.014) and semirecumbent (*P* = 0.038) positions, and the values measured at peak exercise (*P* < 0.0001) (Fig. 4). The relationship between percentage tracheal collapse measured during MRI and bronchoscopy evaluations are displayed in Fig. 5.

### Tolerability and Safety Findings

No serious adverse events were reported. There were no episodes of dysrhythmia nor exercise-induced syncope. One individual developed bronchoconstriction following CBE, with a forced expiratory volume in one second (FEV_1_) reduction of 11%; this returned to baseline values following β-2 agonist administration. In all other cases, there was no minimal reduction in FEV_1_ (*P* < 0.89) following CBE. There was no incidence of hemoptysis.

The most common adverse event reported in 10 (40%) subjects was sore throat/hoarse voice, followed by three (12%) cases of epistaxis and three (12%) who reported transient voice changes lasting <24 h. Two (8%) subjects reported nasal discomfort, and two (8%) presented with a mild fever postprocedure. There was a singular report of postprocedural cough lasting 72 h. The majority (64%) of adverse events were resolved within a 24 h period. Two subjects reported a sore throat for the subsequent 96 h.

Postprocedural feedback identified that 64% of the cohort reported that topical anesthetic caused discomfort, and almost half found placement of the bronchoscope caused discomfort. Only 20% reported discomfort associated with the camera during the exercise component of CBE. In addition, only 8% reported discomfort associated with exercising in the specialist headgear.

## DISCUSSION

**Figure 6.**
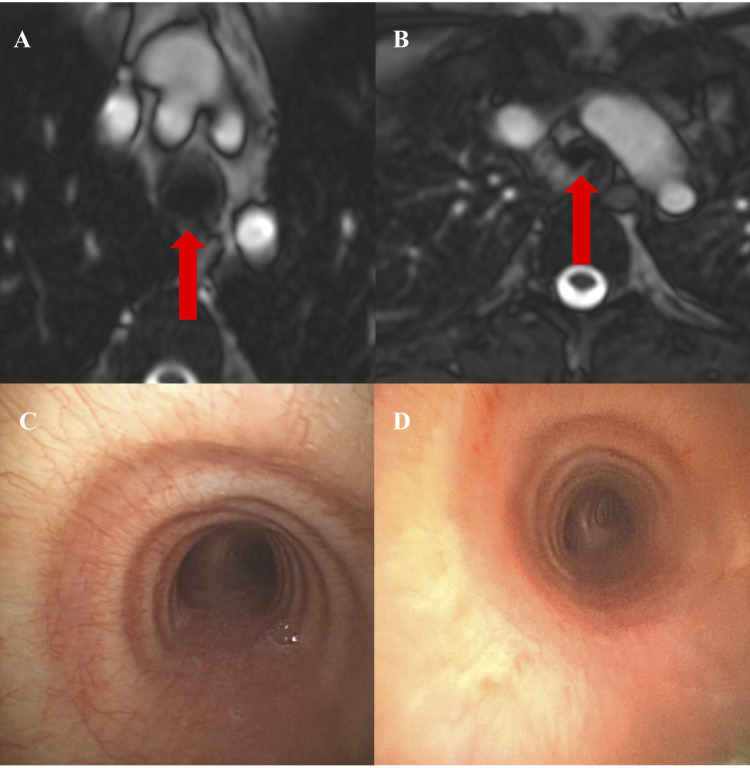
*A*–*D*: dynamic expiratory collapse in a 27-yr-old male subject. Paired axial magnetic resonance imaging (MRI) images [inspiration panel (*A*) and expiration panel (*B*)], approximately 1 cm above bifurcation, showing 83% expiratory reduction in tracheal luminal cross-sectional area (arrow). *C*: diagnostic bronchoscopy of same subject, resting airway inspection. *D*: max expiratory phase closure evident on bronchoscopic images obtained at peak exercise.

Flexible bronchoscopy during vigorous exercise permits visualization of the tracheobronchial tree during real-life, ambulatory-based physical activity in healthy, nonsedated individuals. In this study, the CBE assessment provided novel insight regarding the structural behavior of the trachea and main bronchi during vigorous exercise. Accordingly, in a cohort of healthy subjects, of normal body mass, we found relative stability of the large airway during this type of ventilatory challenge, with no evidence of significant large airway collapse or EDAC (Fig. 6). In contrast, when large airway movement was assessed via a forced expiratory maneuver, using MRI, EDAC was present in over half (64%) of subjects. These findings, thus, present novel insight regarding the physiological behavior of the large airways during the hyperpnea arising from physical activity, and also challenge the role of static forced maneuvers being used as a surrogate to characterize large airway behavior in this context.

In the evaluation of any propensity to large airway collapse, there is continued debate regarding the gold standard modality used to assess large airway caliber. Bronchoscopy has long been considered the “gold standard” assessment by clinicians. However, Mitropoulos et al. (18) revealed an imaging-based investigation strategy has been used or favored in ∼80% of characterizations reported in the literature. The reason for the discrepancy in the prevalence of EDAC observed between bronchoscopy and MRI assessment in this study is not clear. Particularly, as the exaggerated tracheal collapse findings in some of the symptom-free subjects would be considered clinically significant. These findings could relate to alterations in maximal force generation due to the presence of an invasive modality that acts to splint the airway, preventing some degree of airway collapse. Currently, there is a lack of evidence to determine the best method of large airway assessment, and further study is required. The CBE approach aims to provide real-life measurements in the context of incremental-based exercise to voluntary exhaustion, and it appears that findings observed during forced expiratory assessment on MRI differ from the CBE approach, at least in healthy people of normal body mass.

The physiological mechanisms that control large or central airway behavior during vigorous breathing remain to be determined. Collapse or inward movement of the large airways is a prevalent finding in individuals with underlying airway diseases such as chronic obstructive pulmonary disease (COPD) (19), asthma (20), or in obese individuals (21). There are various physiological models that are proposed to explain EDAC, including the equal pressure-point (EPP) theory (1). Intraluminal pressure decreases as air flows from the distal airways to the proximal airways. During forced expiration, an EPP will occur at the point where intraluminal and extraluminal pressure become equal, dividing the airways into upstream (distal) and downstream (proximal) segments. In the upstream segment, transpulmonary pressure is positive, and thus, keeps the airway open; unlike in the more proximal airways, where transpulmonary pressure is negative and collapse may occur. In a clinical population, damaging pathology (e.g., inflammation) has the potential to increase distal airway resistance, impair the structural cartilaginous integrity, and alter the smooth muscle tension of the large airway wall. In this context, where airway stability may be lost, there are changes to the length of the flow-limiting segment that may result in the earlier formation of an EPP. As a consequence, the tracheobronchial tree may become more prone to excessive collapse (1).

The impact of evidence of large airway collapse on exercise capability is not clear. In a cohort of individuals with COPD of varying degrees of airflow obstruction, Boiselle et al. (4) revealed no relationship between excessive tracheal collapse and measures of physiological function, including pulmonary function and 6-min walk test distance. This provides some evidence that incidental findings of exaggerated central airway collapse may not impair functional capacity and exercise tolerance. Certainly, the ventilatory demands of physical activity are different from that of forced expiratory maneuvers, with obvious, and more marked differences in changes in the intraluminal pressure gradient associated with the rapid, sudden exhalation that occurs during forced expiratory maneuvers. It may, therefore, not be surprising that we observed a difference in the maintenance of large airway patency on exercise when compared with forced expiratory maneuvers.

The ability to visualize large airway movement with CBE may provide important insight into the evaluation and work-up of individuals with exertional, unexplained dyspnea or in clinical populations with an established diagnosis, but refractory exertional symptoms. To date, there are very few reports of the use of exercise bronchoscopy in this context. Weinstein et al. (22) detailed a case series evaluating EDAC with exercise bronchoscopy in six young, healthy military personnel presenting with exertional symptoms post deployment. Conventional pulmonary investigations such as spirometry, bronchoprovocation testing, and cardiopulmonary exercise testing did not provide insight. It was argued that the use of real-time bronchoscopic visualization, in this context, provided functional and anatomical assessment at a time commensurate with exertional symptoms, which permitted diagnosis of EDAC.

Currently, treatment options for EDAC-associated exertional symptoms are limited. The use of continuous positive airway pressure (CPAP) during exercise may act as a “pneumatic stent” that permits maintenance of airway patency and overcomes expiratory collapse in patients with EDAC (23). In this context, CPAP has been used anecdotally to provide therapeutic symptom relief in cases of tracheobronchomalacia and EDAC (24). Furthermore, ambulatory CPAP use has been associated with an improvement in 6-min walk test distance, and accompanying reductions in respiratory neural drive (25). Future work is needed to explore the impact of CPAP on exercise tolerance and physiological response in patients with EDAC in formal, pragmatic settings, with CBE potentially providing useful insight in this context. An alternative treatment approach in severe cases of large airway collapse is mechanical airway stenting and tracheobronchoplasty. These procedures aim to reinforce and stabilize the posterior membranous portion of the airway and have been demonstrated to provide both symptomatic and objective improvements (26).

Our results are not without safety considerations and postprocedural adverse events. The report of adverse events appears to be higher than comparator data from large data series, reporting the risk associated with conventional bronchoscopy in clinical populations (27). Given the fact that vigorous exercise was undertaken, we might expect a different risk profile from findings in the resting setting, and a key aim of our work was to capture adverse events carefully, and highlight associated risks to help modify the CBE protocol in future work. The prevalence of abnormal physiological parameters during flexible bronchoscopy procedures is likely to be different when adverse event collection periods vary, with data collection in some case series occurring for 2 h postprocedure (27). It is challenging therefore to directly compare event rates with those captured by a dedicated physiologist tasked with observing a novel intervention. The sensitivity of continuous monitoring, as conducted during the CBE assessment, could be considered to be higher than the periodic performance of intraprocedure monitoring. The importance of close cardiovascular monitoring and photoplethysmography monitoring during the CBE procedure should not be understated. We report three individuals for whom exercise was terminated due to elevated systolic blood pressure. An arbitrary systolic blood pressure cut-off value of 225 mmHg was applied as a pragmatic and cautionary value in this study. This differs from the widely accepted recommendation of exercise termination once a systolic blood pressure of 250 mmHg is met (28). If these blood pressure criteria were used, only one individual would meet the criteria for exercise termination, and the nonreporting of the remaining two “hypertensive” events could be considered. It is challenging to compare these findings with previous studies, as the precise prevalence of an exaggerated blood pressure response (i.e., >210 mmHg on vigorous exercise) in healthy participants varies but has been reported as high as 42% in one study (29). Regardless, the elevated blood pressure findings prompted the investigators to terminate exercise, and in all cases, the blood pressure returned rapidly (<3 min) to preexercise values, upon exercise cessation.

During exercise, findings of significant oxygen desaturation in healthy individuals are uncommon, although the phenomenon can be seen in athletic individuals to some degree, and without obvious cause in exercise-induced arterial hypoxemia (30). Desaturation is a recognized risk of bronchoscopy but is most typically reported in sedated individuals. In our cases, subjects rapidly resaturated upon exercise termination, within 1 min. Currently, the mechanisms for the transient desaturation observed in two subjects are not clear, but we speculate the presence of the bronchoscope may have resulted in relative hypoventilation, transiently impacting gas exchange. Further study is required to explore the impact of the bronchoscope on exercise ventilatory mechanics, breathing patterns, and subsequent influence on pulmonary hemodynamic fluctuations, with the need for a cautious approach apparent.

The study assesses the feasibility of achieving a maximally exhaustive state in healthy subjects undergoing bronchoscopy. Should such a technique be used clinically, a submaximal approach, or an exercise intensity matched to that during which a patient reports exertional symptoms occur, could be considered sufficient for diagnostic purposes. An adapted exercise protocol or cessation at the point of diagnostic determination could also be considered as a more cautious approach. The BORG dyspnea findings recorded in this study are in keeping with those expected at the end of exhaustive exercise (31). However, further study is required to evaluate the potential differences in perceptual response of bronchoscopy during vigorous exercise.

In keeping with previous findings, we observed declines in postprocedural spirometry, which may be attributed to administration of topical lignocaine (32, 33). Sufficient topical anesthesia was applied to the upper airway and the tracheobronchial tree as a prerequisite for the successful performance of the bronchoscopy assessments. With respect to the impact of the anesthetic application, Baier et al. (34) reported the use of topical lidocaine did not alter respiratory resistance, however, the impact on exercise performance within our study is not known and requires further evaluation in future studies.

It is of note that findings of mild exercise-induced bronchoconstriction (EIB) are recognized to occur in previously healthy individuals (35), and as such, the single case of a significant fall in FEV_1_ may not be procedurally related. With respect to the tolerability of the exercise component, it was clear that the majority of the subjects reported discomfort related to the initial passage of the scope, and not from the addition of the exercise protocol. It is not clear that we can compare our tolerability findings to those of patients, given the lack of sedation and fact that more general data arises from patients undergoing this test for a clinical indication. We believe that refinements to the application and delivery of the topical anesthetic will further improve tolerability.

### Methodological Considerations

There were several methodological considerations for this study. The cohort studied was a young, low BMI, athletic population, which makes application of findings to cohorts with different characteristics difficult to apply. This may be of particular importance in cohorts where EDAC prevalence is greater, such as those that are older, present with pulmonary comorbidities, and/or a high BMI (21).

Second, no ventilatory data was obtained during the exercise component of the bronchoscopy, therefore, the physiological end point of the exercise test was solely based on peak HR achieved. Such an approach fails to consider peak oxygen uptake plateau or other markers of maximal effort, such as respiratory exchange ratio and peak minute ventilation as a percentage of maximal ventilation. However, as this is an initial, early phase study, there are currently no prospective data evaluating the effects of a bronchoscope in situ on airflow, ventilatory mechanics, and/or breathing patterns during exercise. Future work will aim to combine airway visualization and the capture of ventilatory data +/− esophageal pressure measurements, as successfully demonstrated at the laryngeal level (36). In this context, there will be additional value in the concomitant evaluation of cardiac and pulmonary disease, or to provide reassurance in their absence, as well as preventing unnecessary subsequent investigations and testing.

It is conceivable that the testing methodology used, in particular placement of a bronchoscope acts to “splint” the airway and provide some form of auto-positive end-expiratory pressure (PEEP) effect, preventing some degree of expiratory collapse. Dynamic laryngeal narrowing during expiration has been observed in patients with COPD, and it is postulated that the movement of the upper airway aperture may preserve a balance between optimal expiratory flow and used as a mechanism to offset PEEP (37). As mentioned earlier, further work is needed to evaluate pressure effects during exercise with direct intraluminal pressure measurement.

In addition, a factor to consider is the effect of bronchoscope placement on breathing resistance. The placement of a bronchoscope creates a narrowing or resistance relative to its diameter, along the length of the airway segment it occupies. This may influence airway stability and forces favoring large airway collapse. As such this may partially explain the difference in prevalence seen with bronchoscopy when compared with MRI. However, the caliber of bronchoscope occupies a small fraction of the airway lumen, and the fact that there was evidence of excessive tracheal collapse in some individuals during resting forced maneuvers, implies that this effect is likely to be limited, and does not confound the differences we describe between exercise and the resting bronchoscopic states.

Nevertheless, although we aimed to compare a novel airway visualization approach against established EDAC assessment methods performed at rest, the comparisons of EDAC between these modalities are limited given the differences in methodologies. However, forced expiratory maneuvers used during bronchoscopy and/or imaging techniques are used in routine clinical practice, and thus, it is relevant that our findings reveal a difference compared with what occurs during exercise hyperpnea. Further study of the impact of exercise on airway collapse with imaging modalities (e.g., MRI with exercise) would be informative, in this respect.

We opted to use a treadmill exercise test aiming to achieve the highest possible ventilatory effort/peak oxygen uptake, specifically, to characterize large airway behavior and propensity to collapse during real-world, ambulatory exercise. However, we do recognize the use of alternative modalities of achieving exercise hyperpnea (i.e., cycle ergometry) (28) or carbon dioxide-induced hyperpnea for this type of assessment (38). Further prospective study is required to determine the feasibility of alternative modalities of hyperpnea in this context and setting.

Another consideration when using the CBE approach is to determine the potential impact of any discomfort arising from the headgear when exercising. The majority (92%) of the cohort reported no discomfort associated with wearing the headgear, and the two individuals that did, commented on the tightness of the straps, rather than the burden of the weight of the equipment. The impact and discomfort of carriage of a bronchoscope in headgear would need to be considered carefully in cases where head, neck, or thoracic cage pathology were reported. As a similar equipment setup is frequently used during conventional treadmill CLE testing, and the weight of the plastic disposable bronchoscope and tubing is small, such equipment may not impact significantly on exercise findings.

Our study was designed to limit the number of forced maneuvers performed by subjects; however, insight may have been provided by asking subjects to perform an additional forced expiratory maneuver in a standing position with a bronchoscope in situ. This maneuver would act as a control for the exercise measurements and provide a comparison of tracheal movement in a similar body position, and thus, should be considered in future studies.

In the assessment of EIB, subjects performed spirometry at only one time point, within 5 min of exercise cessation, which may have reduced the sensitivity to detect EIB. Our subjects were healthy and reported no postexertional dyspnea or chest tightness; however, in future studies, serial spirometry measurements performed as per the ATS EIB guidelines (39) may ensure the accurate assessment and detection of EIB.

Currently, tracheal lumen collapse during bronchoscopy is classified using crude, arbitrary, and subjective scores, which may be prone to differences in clinician agreement. A more robust and objective classification scoring system is required in future studies. With progress and development in machine learning, artificial intelligence software, and other digital tools, computer-generated scoring systems based on surrounding tracheal structures may improve objective quantification and enhance the accuracy and reliability of scoring.

In conclusion, in a cohort of healthy individuals, continuous bronchoscopy permits direct visualization of the tracheobronchial tree and any tendency to tracheal luminal collapse. In this cohort, there was no evidence of EDAC observed at peak exercise, despite evidence of this phenomenon on forced expiratory maneuvers on imaging and standard bronchoscopy. Our findings highlight the importance of careful monitoring of oxygen saturation and blood pressure during CBE, and future work should focus on consideration of how tolerability may be improved. Future research should also aim to assess tracheal movement in a clinical population with EDAC and to assess ancillary parameters or exercise performance, gas exchange, and pressure changes, to permit the combined characterization of exercise-induced changes in tracheal movement and the associated physiological impact.

## APPENDIX

Subjects completed a postexercise tolerability questionnaire that provided feedback on the CBE assessment as displayed in Fig. A1.

## DATA AVAILABILITY

Data will be made available upon reasonable request.

## GRANTS

The RELACS charity and the Royal Brompton Hospital Charity funded the study and Z. J. Williams’ salary.

## DISCLOSURES

No conflicts of interest, financial or otherwise, are declared by the authors.

## AUTHOR CONTRIBUTIONS

M.I.P. and J.H.H. conceived and designed research; Z.J.W., C.M.O., J.L.G., L.T.C., and A.T. performed experiments; Z.J.W., J.L.G., and T.S. analyzed data; Z.J.W., C.M.O., J.L.G., T.S., and J.H.H. interpreted results of experiments; Z.J.W. prepared figures; Z.J.W., C.M.O., and J.H.H. drafted manuscript; Z.J.W., C.M.O., J.L.G., L.T.C., P.L.S., M.I.P., T.S., and J.H.H. edited and revised manuscript; Z.J.W., C.M.O., J.L.G., L.T.C., A.T., P.L.S., M.I.P., T.S., and J.H.H. approved final version of manuscript.
